# Effects of the antibiotic rifaximin on cortical functional connectivity are mediated through insular cortex

**DOI:** 10.1038/s41598-021-83994-4

**Published:** 2021-02-24

**Authors:** Davide Sometti, Chiara Ballan, Huiying Wang, Christoph Braun, Paul Enck

**Affiliations:** 1 MEG-Center, University of Tübingen, Tübingen, Germany; 2 Hertie Institute for Clinical Brain Research, University of Tübingen, Tübingen, Germany; 3 DiPSCo, Department of Psychology and Cognitive Science, University of Trento, Rovereto, Italy; 4 AAK, Department of Special Nutrition, AAK China Ltd, Shanghai, China; 5 CIMeC, Center for Mind/Brain Research, University of Trento, Trento, Italy; 6 Department of Internal Medicine VI, University Hospital, Tübingen, Germany

**Keywords:** Gastroenterology, Neuroscience, Social neuroscience, Stress and resilience

## Abstract

It is well-known that antibiotics affect commensal gut bacteria; however, only recently evidence accumulated that gut microbiota (GM) can influence the central nervous system functions. Preclinical animal studies have repeatedly highlighted the effects of antibiotics on brain activity; however, translational studies in humans are still missing. Here, we present a randomized, double-blind, placebo-controlled study investigating the effects of 7 days intake of Rifaximin (non-absorbable antibiotic) on functional brain connectivity (fc) using magnetoencephalography. Sixteen healthy volunteers were tested before and after the treatment, during resting state (rs), and during a social stressor paradigm (Cyberball game—CBG), designed to elicit feelings of exclusion. Results confirm the hypothesis of an involvement of the insular cortex as a common node of different functional networks, thus suggesting its potential role as a central mediator of cortical fc alterations, following modifications of GM. Also, the Rifaximin group displayed lower connectivity in slow and fast beta bands (15 and 25 Hz) during rest, and higher connectivity in theta (7 Hz) during the inclusion condition of the CBG, compared with controls. Altogether these results indicate a modulation of Rifaximin on frequency-specific functional connectivity that could involve cognitive flexibility and memory processing.

## Introduction

The interest in human gut microbiota and its effects on CNS functioning increased exponentially over the last decade^1–9^. Strong preclinical evidence testified an effect of the GM on brain functioning and development^10^. Following the pioneering study of Sudo and colleague^11^ reporting an alteration of the hypothalamic–pituitary–adrenal (HPA) stress response in germ-free mice, data have accumulated confirming the role of the commensal microbiota in stress responsiveness^12–15^, anxiety and depression-like behaviors, and cognitive functions^16–19^. However, when studies are translated into humans, understanding the effects of GM on CNS becomes more difficult^20^. Significant ethical constraints and several environmental factors such as diet^21,22^, psychological stress^23^, tobacco and alcohol consumption^24,25^ limit the causational interpretation of the interaction between GM and brain functioning. Nevertheless, the approach of targeting human GM^26–28^ has offered a potential method to investigate its effect on the CNS.

Starting from the idea of a possible application of probiotics as adjuvant therapy for the treatment of the major depressive disorder^29^, the curiosity in the effects of some specific “*Psychobiotic*”^30^ influencing the functionality of the central nervous system has grown rapidly^31–35^. Clinical studies reported alteration in cognitive performance and self-reported measures of stress, anxiety, and depression-like behavior^36–39^, following *Lactobacillus* and *Bifidobacterium* strains intake in healthy volunteers. Neuroimaging methods offered a deeper understanding of the effects of probiotics on brain activity. Tillisch and colleagues^40^ demonstrated that 4-weeks consumption of a fermented milk product containing probiotics altered the activation of interoceptive and somatosensory regions, together with rs midbrain connectivity, in response to an emotional attention fMRI task. Reduced limbic reactivity to fearful faces was found in patients with Irritable Bowel Syndrome (IBS) after 6-weeks of treatment with a *Bifidobacterium* strain^41^. Other neuroimaging studies reported alteration in brain activity associated with emotional decision-making and memory processes^42^, rs functional connectivity^43^, and stress-related electrophysiology^44,45^, following *Bifidobacterium* and *Lactobacillus* intake by healthy volunteers. Nonetheless, despite these encouraging results, not all potential probiotic interventions that had shown an effect on CNS in preclinical studies have translated successfully in humans^46^. Thus, highlighting the need for a more comprehensive understanding of the role played by the intestinal microbiota in altering brain activity.

A complementary approach to the study of GM-fc interaction by administering probiotics is offered by antibiotics, which typically causes a decrease in microbiota diversity^47–49^. Preclinical studies have repeatedly shown the impact of antibiotic-induced GM depletion on stress-related behavior and cognitive functions in rodents^50–54^. Concerning human studies on GM, antibiotics have almost exclusively been studied to test their efficacy in treating gastrointestinal (GI) diseases^55,56^. Among the different antibiotics employed, rifaximin has been proven effective in treating small intestine bacterial overgrowth (SIBIO—review^57^) and GI diseases such as traveler’s diarrhea, functional bloating, and irritable bowel syndrome (IBS). Furthermore, due to its negligible systemic absorption after oral administration (less than 0.4%, reviews^58,59^), the risk of developing drug resistance in healthy subjects is minimized^60–63^. For these reasons, Rifaximin has been recently selected by our group to first examining the effects of antibiotics on CNS in healthy volunteers^64^. The study investigated the effects of 7-weeks Rifaximin intake on brain activity with MEG. Results for antibiotic treatment displayed an increase of self-reported emotional well-being, together with an alteration in resting-state alpha and task-related beta power associated with a stress-reducing effect. These first findings exhibited an effect of Rifaximin on brain activity similar to the one exerted by the intake of probiotics^45^. Nevertheless, whether Rifaximin may modulate intracortical coupling, thus altering functional connectivity, is yet unknown.

Herein, we aimed at expanding our previous findings on spectral power investigating the effect of 7-days Rifaximin administration on MEG functional connectivity in healthy subjects. Brain connectivity was investigated during rest and during a social stress-inducing paradigm (Cyberball game—CBG) eliciting feelings of ostracism. We anticipate the involvement of the insula in signalling Rifaximin effects to the cortex, due to its critical role in interoception^65,66^. Considering the presence of visceral afferent projections that from the vagus nerve reach the insular cortex through the thalamus, the insula seems a plausible candidate to be a significant hub within the microbiota-gut-brain axis. Given the pilot nature of the study, no preconceived assumptions regarding the different frequencies of coherence that might be affected by the treatment, were present. Inclusion criteria, group characteristics, and experimental paradigm have already been reported in detail^64^ and will not be reported here unless necessary for the understanding of the current approach.

## Results

### Effects of rifaximin on resting-state connectivity

**Figure 1:**
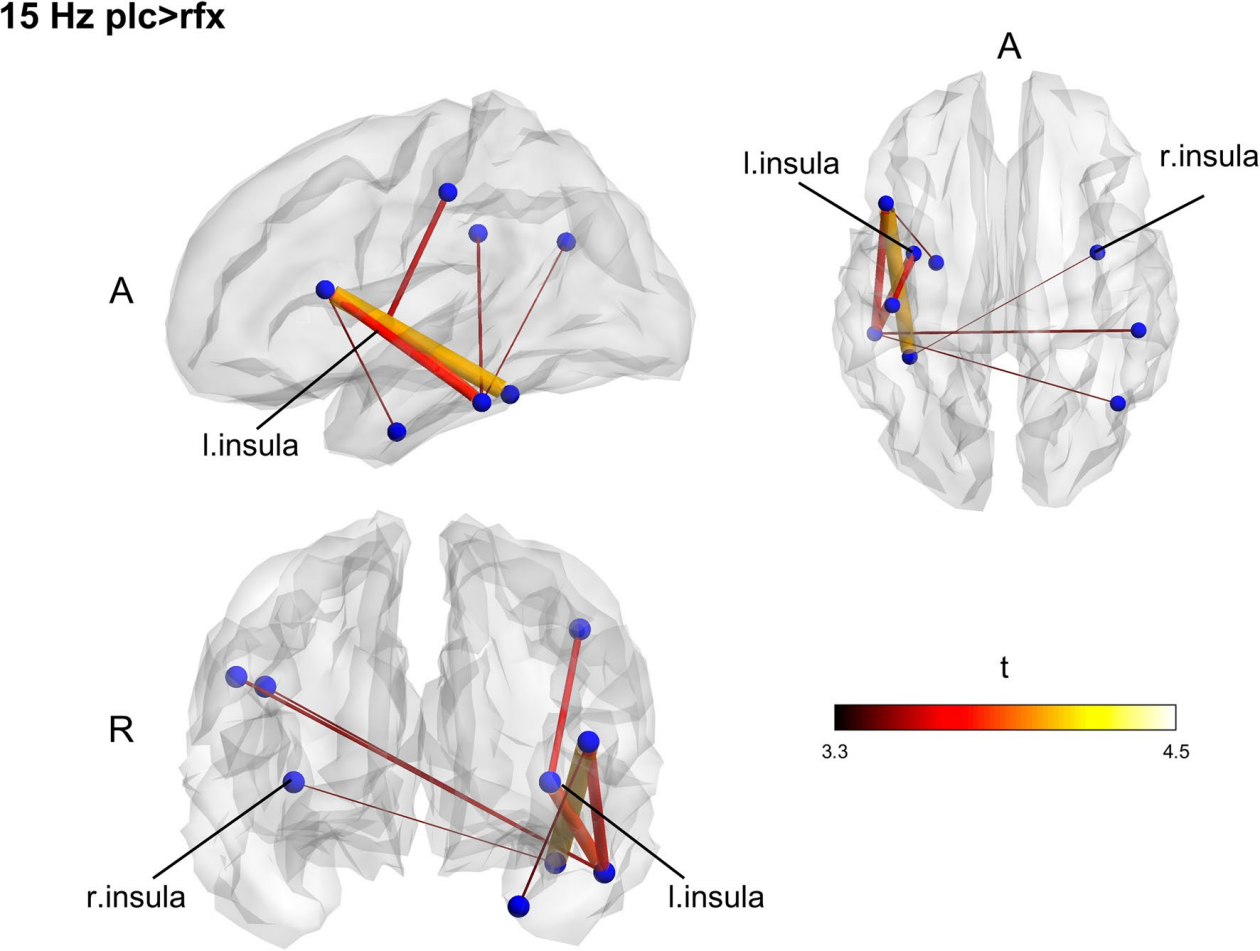
Between groups functional connectivity comparison in post-treatment session. The placebo group displays two networks with increased connectivity in slow and fast beta band (15 Hz) compared to the Rifaximin group. Clusters of higher connectivity are located mainly in the left hemisphere, involving frontal, temporal, and parietal areas. To note the presence of the insular cortex within the networks. Color and size of the edges are proportional to t-statistic (t) value (*A* anterior, *R* right).

**Figure 2:**
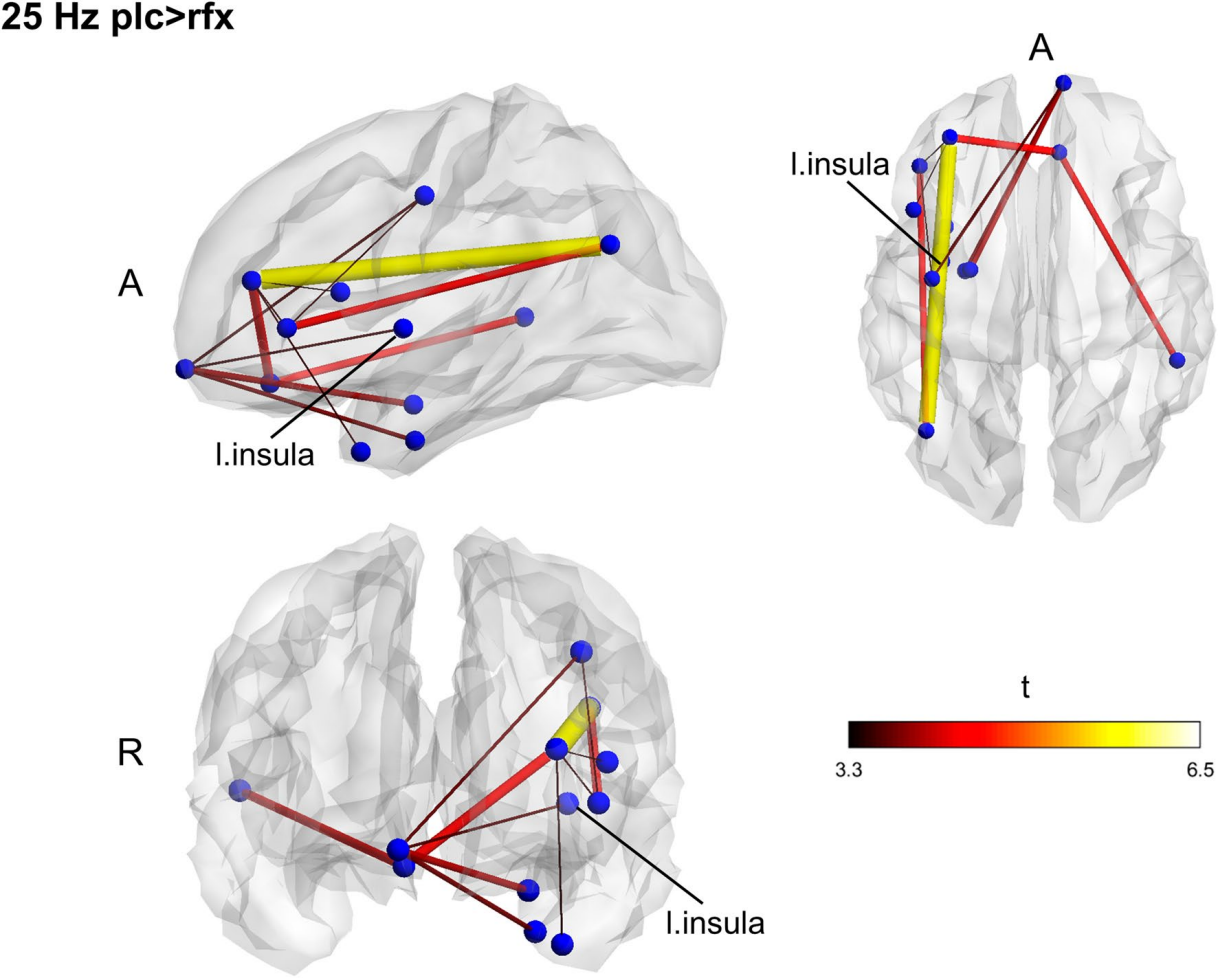
Between groups functional connectivity comparison in post-treatment session. The placebo group displays two networks with increased connectivity in slow and fast beta band (25 Hz) compared to the Rifaximin group. Clusters of higher connectivity are located mainly in the left hemisphere, involving frontal, temporal, and parietal areas. To note the presence of the insular cortex within the networks. Color and size of the edges are proportional to t-statistic (t) value (*A* anterior, *R* right).

To check the presence of significant differences regardless of the treatment, we compared the groups at the baseline. No differences were found in any frequency band. After 7-days treatment, the placebo group displayed networks with higher fc in slow-beta (15 Hz; *p* = 0.045) and fast-beta (25 Hz; *p* = 0.026) (Figs. 1, 2). Contrasting pre-and post-intervention within the groups, we tested whether post-treatment differences were ascribed to increased fc for the placebo group or decreased connectivity in the Rifaximin group; however, no significant differences were found.

## Effects of rifaximin on functional connectivity during CBG

**Figure 3:**
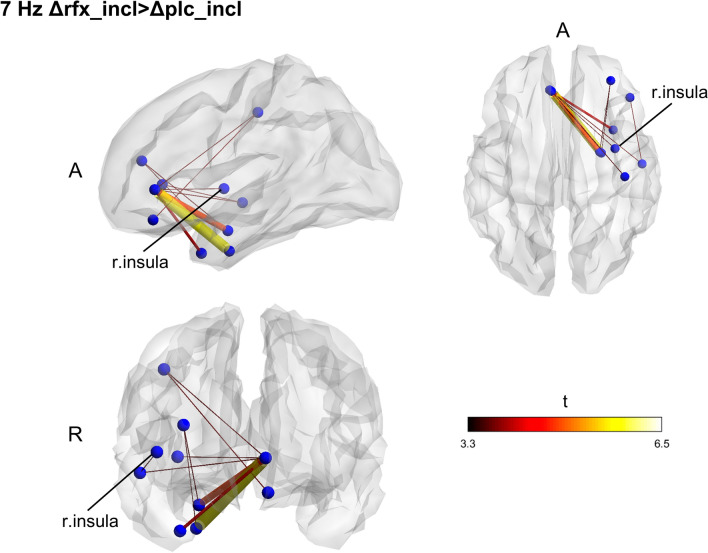
Between groups functional connectivity comparison during CBG in post-treatment session. Theta (7 Hz) higher fc connectivity network found in Rifaximin group during the inclusion condition. The cluster of higher connectivity involves the frontal and temporal lobe, mainly in the right hemisphere. The right insular cortex is highlighted. Size and color of the edges are proportional to the t-statistic (t) value (Δ = post–pre matrices, *A* anterior, *R* right).

**Figure 4:**
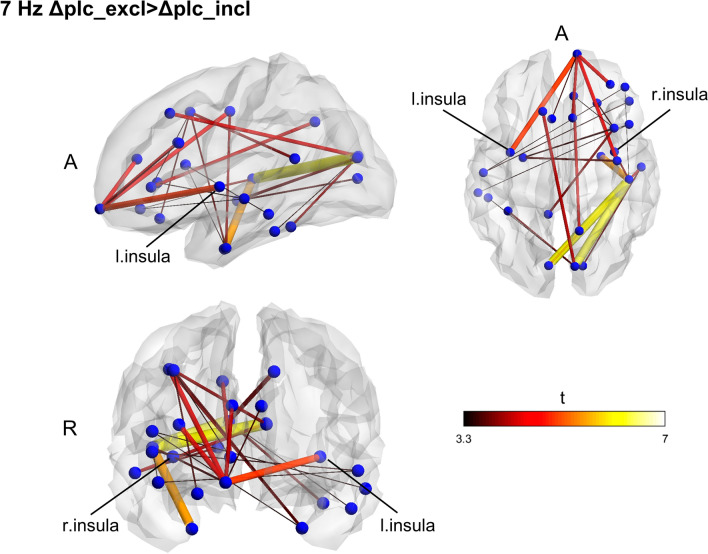
Within groups, functional connectivity comparison between exclusion and inclusion condition in post-treatment session. Placebo group (Δplc) displays a higher functional connectivity network in theta during the exclusion condition of the CBG as compared to the inclusion one. The identified cluster widely extent across all the brain, involving frontal, parietal, temporal, and occipital areas. To note the presence of the insula again, size and color of the edges are proportional to the t-statistic (t) value (Δ = post–pre matrices, *A a*nterior, *R* right).

**Figure 5:**
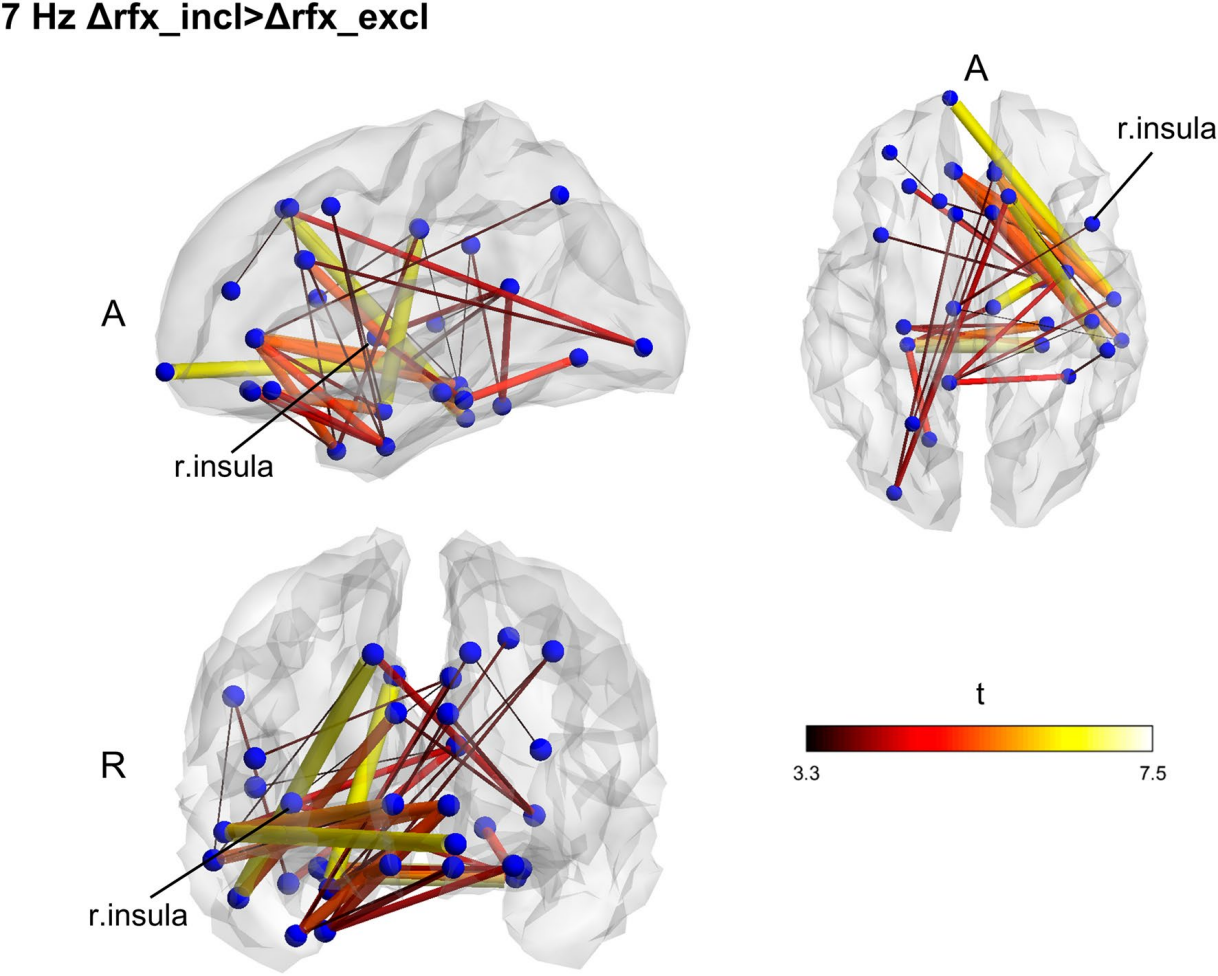
Within groups, functional connectivity comparison between exclusion and inclusion condition in post-treatment session. An opposite pattern was found for the Rifaximin group (Δrfx), displaying a higher functional connectivity network in theta during the inclusion condition. Again, the network is largely widespread across the brain. To note the presence of the insula again, size and color of the edges are proportional to the t-statistic (t) value (Δ = post–pre matrices, *A a*nterior, *R* right).

Contrasting exclusion and inclusion conditions at the baseline for all subjects, we found no significant effect of stress on connectivity in any of the frequencies. Also, no significant differences between the groups were found at baseline. Functional connectivity changes due to the interventions were computed by subtracting the pre-treatment connectivity matrices from the post-treatment ones for each CBG condition and each frequency band and then performing a between-group comparison on the computed matrices. The Rifaximin group displayed a network with higher fc in the theta band (7 Hz; *p* = 0.038) (Fig. 3) during the inclusion condition. Within-group differences between exclusion and inclusion conditions post-treatment were also found, underlining higher network fc in theta during the inclusion condition for the Rifaximin group (*p* = 0.028) and exclusion for the placebo one (*p* = 0.021) (Figs. 4, 5).

## Discussion

The present study investigated the effects of 7-days Rifaximin intake (600 mg/d) on MEG functional connectivity in healthy subjects. A double-blind, placebo-controlled, pre-and post-intervention design was undertaken. Functional connectivity was estimated during rest and during a social-stress-inducing protocol (CBG), using imaginary coherency to quantify the connectivity magnitude between cortical brain regions. The resting-state analysis highlighted higher fc networks in different beta bands (15 Hz and 25 Hz) during the post-treatment session for the placebo group. However, the lack of any significant effect in the within-group comparison between pre-and post-treatment sessions does not allow us to derive any conclusion regarding the directionality of the aforementioned effect. It is plausible that the placebo group slightly increased connectivity between the treatment session, while Rifaximin, on the contrary, slightly decreased it, thus resulting in a significant group difference in the follow-up session. No effects of social exclusion induced stress on fc were found, comparing CBG conditions. However, differences in theta (7 Hz) connectivity could be demonstrated between groups following the treatment. The Rifaximin group displayed a network with higher theta fc during the inclusion condition than the placebo group. Within-group comparisons between CBG conditions confirmed the presence of higher theta fc in the inclusion condition for the Rifaximin group after treatment, while the same increase of fc in theta, yet in the exclusion condition, was found for the placebo group. Given the presence of the insular cortex in all the identified networks, group differences in functional connectivity might be mediated by this area in case of alterations of gut microbiota following Rifaximin intake. However, considering the study’s novelty and the current lack of well-founded theories on the effects of antibiotics on functional connectivity, clear interpretations of the results are difficult and, therefore, predominantly of speculative nature. Furthermore, while the involvement of the insular cortex was consistent across both the resting state and the CBG protocol, frequencies of coherence—sensitive to tasks, contexts, and brain states—differed between paradigms.

Coherence here considered as a measure of functional connectivity^67^ has been proposed as a mechanism allowing flexible communication between groups of neurons^68^, subserving the integration of multisensory information and thus allowing for multisensory perception^69^, as well as for our cognitive flexibility^70^. In our study, group differences of coherence levels in beta bands were found after treatment. Coupling in beta-band activity has been proposed to be associated with the maintenance of the status quo, resulting in a strong inhibition of behavioral and cognitive changes during a pathological exacerbation of beta-band coherence^71^. Here, we speculate that lower connectivity found in beta for the Rifaximin group during resting state might indicate an enhancement of cognitive flexibility following GM-antibiotic-alterations. One might argue that the lack of any significance within the effect of treatment for Rifaximin group does not support this interpretation. However, since both the group were exposed to the same variables in terms of time, measurement repetition, and intervention paradigm, it is justified to assume that lower beta networks coherence found in Rifaximin group is due to the active intervention.

Cognitive flexibility is defined as the ability to shift between “cognitive status,” adapting cognitive processing strategies to changes in the environment^72^. Low cognitive flexibility has been associated with greater negative appraisal of stressful situations^73^. Clinically, impairment in cognitive flexibility has been linked to excessive rumination in depression^74^, emotion dysregulation in mood and anxiety^75^, obsessive-compulsive^76^, and eating disorders^77^. Potential evidence supporting the speculation of a GM-cognitive flexibility link are offered by the irritable bowel syndrome. IBS is characterized by a broader group of symptoms that include abdominal pain, diarrhea, and/or constipation^78^, which have been linked with alterations in GM composition^79–86^. This condition often coexists with different psychological disorders^87–89^. Comorbidity between IBS and depression, a mental disorder that is characterized by a strong focus on negative expectations and impaired cognitive flexibility^73,90–92^, is documented^93–95^. Furthermore, increased perseverative error in the Wisconsin Card Sorting Test^96^ has been observed in IBS patient^97,98^, together with altered brain activity in prefrontal areas, insular cortex, and hippocampus^97^. These findings might indicate impairments in cognitive flexibility, thus corroborating the speculation of a possible connection between GM and high cognitive functions, such as mental flexibility.

Regarding the condition-related enhancement in theta coherence, we speculate state-dependent processing of social stress, which is different following placebo or Rifaximin intake. Theta oscillations have been associated with memory processes^99^. In particular, enhanced theta coherence has been repeatedly associated with successful memory encoding and retrieval^100–107^. Since stress exposure has been seen to enhance emotional aspects of episodic memory^108–110^, our condition-dependent theta coherence network enhancement might reflect a bias towards negative (placebo) and positive (Rifaximin) aspects during the consolidation of the experience of stressful situations. In one study^111^, using word-related memory tests, IBS patients displayed a significant bias towards emotionally negative words, the same as displayed in depressed patients. This study highlights the presence of a confirmatory bias for negative material processing in a condition of GM alteration, again suggesting a possible connection between gut microbiota composition, and an alteration in memory function, that might reasonably be reflected by a condition-dependent enhancement of coherence networks. Noteworthy, the improvement of emotional well-being following the intake of Rifaximin reported in our previous study^64^ is well consistent with here proposed interpretation of Rifaximin-induced brain network effects indicating a positive bias during the consolidation of emotional experiences.

Finally, our hypothesis concerning the presence of the insula, given its important role in interoception^65^, has been confirmed in all the highlighted networks, oscillating either in beta or theta frequency. Therefore, we propose the insular cortex to be a critical node in mediating changes in fc following GM alteration. Several mechanisms have been suggested through which GM alterations might affect CNS and cognitive functions, involving neuro-vagal, endocrine, and immune pathways^3,5,6,9^. The insular cortex is known to be the converging point of the spinothalamocortical pathway, which processes and integrates interoceptive information, transmitted primarily through vagal afferents^65^. These observations highlight the relevance of the insula within the microbiota-gut-brain axis and support the hypothesis of an insula-mediated fc alteration following changes in GM composition. In support of our hypothesis, previous MRI studies described structural and functional alteration of the insula in IBS patients^112–116^. One study^117^ reported significantly increased cortical thickness of the right posterior insula in a group of diarrhea-predominant IBS female patients supposedly related with enhanced interoceptive monitoring. Resting-state insula seed-based analysis in female IBS patients^118^ displayed negative fc between dorsal anterior insula, medial prefrontal cortex, and precuneus, which are a key node of the default mode network (DMN), suggesting the role of the insula in modulating intrinsic fc of major networks and in the pathophysiology of IBS. Relevantly, a recent study on tobacco smokers^119^ using seed-based insula analysis at rest showed a middle insular resting-state fc to frontal and cerebellar areas associated with GM structure, bacterial diversity, and genera (*Prevotella* and *Bacteroides*).

With respect to the effects of Rifaximin on human GM and CNS, so far, evidence emerges mainly from studies on pathological conditions. Reasonably, this fact entails strong limitations on the derivation of any conclusion about a causative role of the treatment since any observed outcome might result from an amelioration of the pathological condition, rather than a consequence of a GM-Rifaximin-induced alteration on CNS. Nevertheless, studying the effects of Rifaximin in patients certainly offers an interesting starting point. As yet, Rifaximin has been successfully used in treating IBS^120–124^, and proved to have the greatest effect on IBS symptoms among different other antibiotics^125^. Interestingly, however, in cirrhotic patients with minimal hepatic encephalopathy (MHE), a condition without obvious clinical manifestation characterized by cognitive impairment in attention, vigilance, and integrative function^126^, Rifaximin has been seen to improve cognitive functioning and health-related quality of life^127–131^. To note that in MHE, which has been linked with significant alterations in the gut microbiome associated with cognition^132^, among the different improved cognitive functions, cognitive flexibility^127^ and working memory^131^ have been reported to improve after Rifaximin treatment.

A multifactorial explanation has been proposed to clarify the nature of the aforementioned improvements, highlighting the potential mechanisms through which Rifaximin might modulate CNS functioning. Reduced endotoxemia and altered microbiota-associated metabolic function, with a change in fatty acids production, have been reported following Rifaximin treatment^128^. Furthermore, it seems that the mechanism of action of Rifaximin, despite its role as a gastrointestinal-targeting antibiotic, might extend beyond the GM, modulating the production of inflammatory cytokines and intestinal permeability^133^. Finally, Rifaximin treatments have been seen to promote beneficial gut bacteria such as *Bifidobacteria* and *Lactobacilli* in patients with gastrointestinal and liver disorders^134,135^. Based on these observations, it is likely that Rifaximin intake might also alter brain functional connectivity in healthy subjects through its focused and yet broad actions on GM and the gastrointestinal tract.

Although the results displayed the potential effects of Rifaximin-induced GM modulation on CNS connectivity, the study presents several limitations. First, in reason of the pilot nature of the study conceived to explore the feasibility of the approach used to generate the hypothesis to be tested in further experiments, only 16 participants were recruited. Therefore, the here presented preliminary results need to be replicated and validated. In addition, the absence of significant differences in age between the groups is most likely due to the large variability of the participants’ ages rather than to a close match of the groups. In future studies, it is highly recommended to better control for age and other factors such as personality traits to increase the specificity of the interpretation of the results. Second, unlike previous investigations using CBG as a social-stressor protocol^136,137^, no evidence signaling the involvement of different networks for exclusion and inclusion conditions was found in the baseline. The reason for the difference is unclear and entails some limitations for the interpretation of the results in the post-treatment session. It is possible that during the baseline session, all the participants were under the same level of stress/excitation regardless of the condition due to the unfamiliarity with the experimental situation, resulting in a flattening of any significant difference in fc. In a future study, it might be reasonable to introduce a task familiarization session before the beginning of the experiment in order to avoid an unfamiliarity effect that could overwrite any Rifaximin and GM-induced fc difference. Nonetheless, this was the first study investigating the impact of social stress on MEG fc, making it difficult to compare our results with previously published work using fc MRI^136,137^. Finally, again in reason of the exploratory nature of the study, no stool samples for microbiome analysis and additional peripheral physiological parameters had been collected. The lack of stool sampling, to include in further research, prevent any clearer conclusion about GM alterations that could have helped to shed light on the mechanism of action through which Rifaximin might affect brain fc. Regarding the physiological parameters, the stress effect of the CBG and the health status of the participant have here been investigated only through self-reported questionnaires^64^. In further investigation, physiological and/or hormonal responses should be considered to increase the objectivity of the results.

In conclusion, our findings showed widespread functional connectivity alterations during rest and during a stress paradigm, following 7-days Rifaximin intake. The presence of the insular cortex in all the highlighted clusters might indicate its potential role in mediating changes in fc, signaling commensal microbiota alterations. Connectivity in beta and theta frequencies was affected, suggesting possible relations with higher cognitive function, such as cognitive flexibility and memory encoding. Further studies are necessary to replicate the results and confirm these hypotheses. A cognitive task-based multimodal neuroimaging study investigating precisely flexibility and memory functioning should be addressed. A larger sample and the inclusion of other test groups (e. g., probiotic, and IBS patients) might offer a more global picture of the mechanism through which GM affects the CNS.

## Methods

### Subjects and study design

Sixteen healthy participants were recruited for the study (9 males; mean age: 27.00 ± 6.39 SD, range 22–49 years of age; BMI: 22.21 ± 1,94 SD). Eight participants completed the intervention with Rifaximin (rfx, 6 males, mean age: 26.50 ± 2.98 SD, range 24–31 years of age; BMI: 22.48 ± 1.64 SD) and eight with placebo (plc, 3 males, mean age: 27.50 ± 8.83 SD, range 22–49 years of age; BMI: 21.94 ± 2.28 SD). Age and BMI were not significantly different between placebo and Rifaximin groups. Exclusion criteria were MEG/MR incompatibility, use of antibiotics within the last 2 months, and psychiatric/gastrointestinal disorder. All participants signed informed consent before joining the study. The protocol has been approved by the Ethics Board of the University of Tübingen Medical School (No. 503/2015BO1, approved on 26.08.2015).

A randomized, double-blinded, placebo-controlled design with pre-and post-intervention assessment was used. All participants underwent two MEG recording sessions, before (baseline—t0) and after 7 days of treatment (t1) either with Rifaximin (3 × 200 mg/d) or placebo pills. During all experimental periods, subjects had to avoid the consumption of probiotic and prebiotic—containing foods. Both groups were tested during rest and while playing a cyber ball game^138^. CBG is an online ball-tossing game in which participants believe that they are playing with two real “others.” In contrast, the dynamics are actually controlled by the experimenter, which can manipulate the inclusion rate of the participant in the game, eliciting interpersonal ostracism, and social stress. In our experiment, the paradigm consists of 4 game sessions, programmed to vary inclusion (incl) and exclusion (excl) conditions. During the inclusion block, each of the players had an equal chance to receive the ball, while during the exclusion block, the “real” participant was largely excluded from the game. The 1/3 of the total 108 trials of the inclusion block, when the participant just observed the virtual players were throwing the ball to each other, were called “not my turns” events. These 36 “not my turn” trials were then compared to the 36 so-called “rejection” trials during the exclusion condition. The beginning of the trial was defined by randomly presenting the ball for 500 to 2000 ms. After one of the players threw the ball, this was moving for 2000 ms before reaching the target player.

### Data recording

MEG data were recorded at the MEG-Center of the Universitätsklinikum Tübingen using a whole-head 275 channels MEG system (CTF Inc., Vancouver, Canada) located in a magnetically shielded room. MEG signals were sampled at 585.94 Hz, with an anti-aliasing filter set at 146.24 Hz. First, 5 min of spontaneous resting-state activity was recorded. Participants were measured in a sitting position and were instructed to move as little as possible, keeping their eyes closed and not to fall asleep. Following the rs session, the CBG paradigm was initiated. Task instructions were projected onto a screen in front of the subject. Participants had to fixate the screen and interact with the game by pressing two different buttons (left and right) of a response box, depending on the direction in which they wanted to throw the virtual ball.

4.3 For the reconstruction of neuromagnetic sources, head anatomical data were acquired using a Siemens Magnetom Trio or Prisma 3 T scanner (Siemens AG, Erlangen, Germany) with a 12-channel head coil. High-resolution (1 mm, isotropic voxels) T1-weighted whole-head structural images were acquired for each participant on different days than the MEG recording. To further coregister the two datasets, we first localized the position of the head during the MEG recording using three fiducial coils (left/right preauricular points and nasion), and we monitored the head motion throughout the experiment with a threshold limit of 5 mm. At the time of the MR scan, we used MRI-visible fiducials, located at the same position as the head coils, to make it easier to localize the correct points further when we realigned the structural images according to the CTF coordinate space of the MEG system.

### Data analysis

MEG data were analyzed using MATLAB (MathWorks Inc., Natick, Massachusetts USA), NBS Connectome^139^, and FieldTrip, an open-source MATLAB toolbox specifically developed for electrophysiological data analysis^140^. BrainNet Viewer^141^ was used for the visualization of the results. Head anatomical data, from MRI acquisition, were processed using Fieldtrip, the software package FreeSurfer^142–144^ and AFNI/SUMA^145–147^.

Continuously recorded data were first filtered to reduce the 50 Hz power line noise using the DFT filter function provided on fieldtrip, which applies a notch filter to the data. Resting-state data were segmented into 150 segments of 2000 ms length. CBG data were segmented in intervals ranging from 1000 ms pre- and 2000 ms post-stimulus onset (triggered by the action of throwing the ball by one of the players) and baseline corrected. Because the CBG paradigm in our experiment alternated inclusion and exclusion conditions in a four games session, we appended all the trials belonging to the same condition. Trials in which the other players threw the ball towards each other during the inclusion blocks were labeled as “inclusion,” and those during the exclusion blocks as “exclusion.” A total of 72 trials for each condition were defined. Next, we manually excluded any trial whose variance exceeded 10^25^ T^2^ in any channel using the *rejectvisual* function provided in FieldTrip. In addition, an independent component analysis to remove non-cortical physiological activity (eye-blink, heartbeat, and muscular components) was performed.

**Figure 6:**
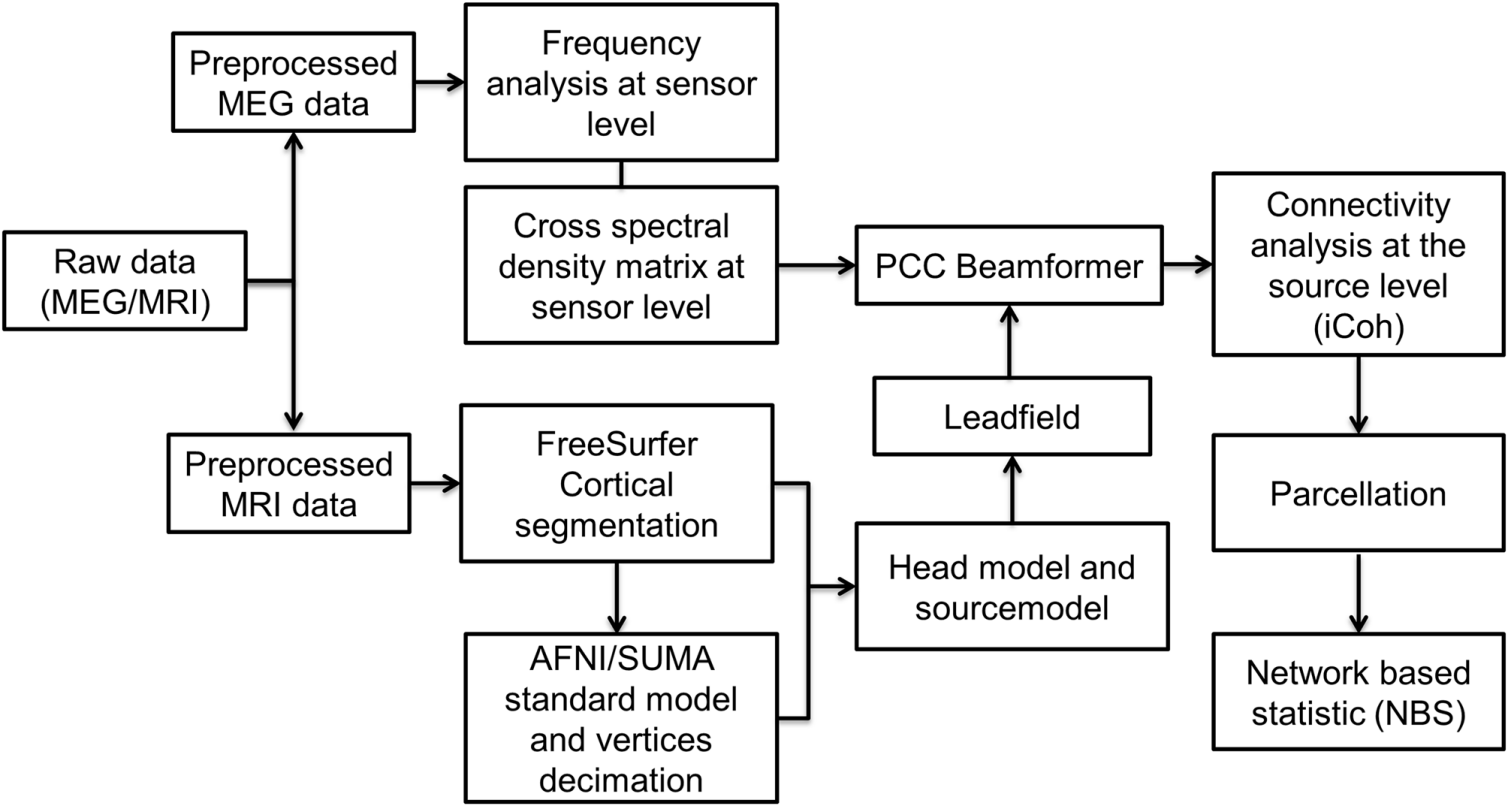
Data analysis pipeline.

For spectrum decomposition and source analysis, we first selected different frequencies of interest, ranging from 5 to 29 Hz, in steps of 2 Hz, with a smoothing window of ± 1 Hz. Then a common spatial filter was computed (independently for resting state and CBG) using partial canonical correlation/coherence (PCC) as a beamformer method^148^. Subsequently, the common filter was applied separately to pre/post and inclusion/exclusion conditions for source reconstruction. This “common filter” approach was used to minimize the possibility of introducing a bias towards one condition during the source estimation, related to differences between the filters themselves rather than an actual difference between conditions. A cortical sheet serving as a source model was used for source reconstruction. In consideration of MEG’s sensitivity mostly to tangential and cortical sources^149,150^, cerebellum and subcortical regions (except bilateral hippocampus and amygdala) were not included in the analysis. All the steps of the pipeline are illustrated in Fig. 6.

In order to co-register the neuromagnetic activities acquired with MEG to anatomical structures, the topology of individual cortices was extracted from individual MRI scans as a three-dimensional mesh. After preprocessing and spectral decomposition, source reconstruction was performed using a cortical sheet with 2004 nodes as source model. Absolute imaginary coherency was used to quantify functional connectivity between nodes of the cortical mesh. Finally, a parcellation scheme was applied to reduce data dimensionality before the statistical analysis.

### Creation of the cortical source model

T1 weighted anatomical images were first realigned according to MNI’s RAS coordinate system and then resliced, defining 1 mm thickness for the slice and specifying 256 × 256 × 256 mm^3^ volume. In addition, a skull stripping of the anatomical image was performed to separate the brain from non-brain tissue using FreeSurfer. We visually inspected the results, manually corrected any error due to too aggressive/conservative skull-stripping, and then proceeded with the automatic segmentation function provided by the software. Each hemisphere was warped into a topological sphere (with white/gray matter boundary defined from FreeSurfer automatic segmentation). Cortex-based inter-subject realignment was done on the warped spheres^143,144^. To decimate and interpolate the vertices generated by FreeSurfer (> 100,000) to 1002 common vertices for the hemisphere, SUMA (Surface Mapper from AFNI suite) was used. This procedure was done to increase source data handling and allow group-level statistical analyses. The same method was applied to the template brain *fsaverage* provided by FreeSurfer to visualize the results further.

### Network analysis

NBS connectome^139^, implementing the nonparametric network-based statistic (NBS) method, was used for the network analysis. The nonparametric way of testing was chosen due to the non-normally distributed values of coherence. Parcellated connectivity matrices were used as input for the analysis. The statistical model was defined in terms of general linear model (GLM), and we chose a test statistic threshold of 3.3 (t). All between and within-group comparisons used either independent or paired sample *t* test, respectively, as a first step of the cluster identification. All tests were corrected for multiple comparisons.

The NBS method involved four different steps. First, it runs a massive univariate test to define a test statistic value for each connection. Second, it compares the t-statistic value of each connection, with the previously defined threshold, identifying all the supra-threshold connections. The next step determines the presence of any topological clusters among the sets of supra-threshold connections. Finally, it permutes the data, repeating the first three steps *n* times (5000 permutations). Data are randomly relabeled depending on the test to be accomplished, involving either the two treatment groups (Rifaximin and placebo), the sessions (pre-and post-treatment), or the CBG conditions (inclusion and exclusion). The size of the largest cluster is recorded after each permutation. Based on all permutations, an empirical null distribution for the size of the largest cluster was generated. The error probability of the observed cluster difference’s error probability was expressed in a one-sided FWER-corrected *p value*^151^ was based on the null distribution.

## Supplementary Information

Supplementary Information 1.
