# Supplementary material for: Effects of the antibiotic rifaximin on cortical functional connectivity are mediated through insular cortex
Source: Sci Rep. 2021 Feb 24;11:4479. doi: 10.1038/s41598-021-83994-4 (PMC7904800; doi:10.1038/s41598-021-83994-4)
Supplement: Supplementary file 1 — Supplementary Information 1. [file 41598_2021_83994_MOESM1_ESM.doc]

**
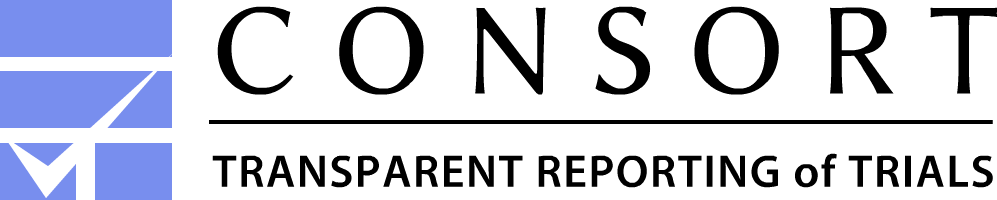
**

**CONSORT 2010 Flow Diagram**

**Allocation**

**Analysis**

**Follow-Up**

**Enrollment**

Assessed for eligibility (n= 26)

Excluded (n= 10 )

♦  Not meeting inclusion criteria (n= 8)

♦  Declined to participate (n= 2)

♦  Other reasons (n= 0)

Analysed (n= 8 )
♦ Excluded from analysis (give reasons) (n=0)

Lost to follow-up (give reasons) (n= 0)

Discontinued intervention (give reasons) (n= 0)

Rifaximin (n= 8 )

♦ Received allocated intervention (n= 8 )

♦ Did not receive allocated intervention (give reasons) (n= 0 )

Lost to follow-up (give reasons) (n= 0)

Discontinued intervention (give reasons) (n= 0)

Placebo (n= 8 )

♦ Received allocated intervention (n= 8 )

♦ Did not receive allocated intervention (give reasons) (n= 0 )

Analysed (n= 8 )
♦ Excluded from analysis (give reasons) (n= 0)

Randomized (n= 16)
